# Early Development of Network Oscillations in the Ferret Visual Cortex

**DOI:** 10.1038/s41598-017-17502-y

**Published:** 2017-12-19

**Authors:** Yuhui Li, Chunxiu Yu, Zhe Charles Zhou, Iain Stitt, Kristin K. Sellers, John H. Gilmore, Flavio Frohlich

**Affiliations:** 1 Department of Psychiatry, University of North Carolina at Chapel Hill, Chapel Hill, NC 27599 USA; 2 Neurobiology Curriculum, University of North Carolina at Chapel Hill, Chapel Hill, NC 27599 USA; 3 Department of Neurology, University of North Carolina at Chapel Hill, Chapel Hill, NC 27599 USA; 4 Department of Cell Biology and Physiology, University of North Carolina at Chapel Hill, Chapel Hill, NC 27599 USA; 5 Department of Biomedical Engineering, University of North Carolina at Chapel Hill, Chapel Hill, NC 27599 USA; 6 Neuroscience Center, University of North Carolina at Chapel Hill, Chapel Hill, NC 27599 USA

## Abstract

Although oscillations during development have been characterized in a wide range of neural systems, little is known about the interaction between these network oscillations and neuronal spiking, and the interactions among different oscillation frequencies. Here we recorded the spontaneous and visual-elicited local field potential (LFP) and multi-unit activity (MUA) in the visual cortex of freely-moving juvenile ferrets before and after eye-opening. We found that both the spontaneous and visually-elicited LFP power was increased after eye-opening, especially in higher frequency bands (>30 Hz). Spike LFP phase coupling was decreased for lower frequency bands (theta and alpha) but slightly increased for higher frequencies (high-gamma band). A similar shift towards faster frequencies also occurred for phase-amplitude coupling; with maturation, the coupling of the theta/alpha/beta band amplitude to the delta phase was decreased and the high-gamma amplitude coupling to theta/alpha phase was increased. This shift towards higher frequencies was also reflected in the visual responses; the LFP oscillation became more entrained by visual stimulation with higher frequencies (>10 Hz). Taken together, these results suggest gamma oscillation as a signature of the maturation of cortical circuitry.

## Introduction

Neural oscillations reflect the functional state of the brain and are thought to dynamically orchestrate brain activity as a function of specific task requirements^1–3^. Oscillations in the developing brain have been studied both in animals using invasive recordings^4–12^, and in humans with electroencephalography (EEG)^13–15^. These rhythms have been implicated in the reorganization and refinement of the neural circuits throughout development^16,17^. In the developing brain, oscillations at different frequencies occur spontaneously^4,5,7,18^ and are also engaged by external stimuli or task demands^4,19^. However, relatively little is known about the interaction between LFP oscillations and spiking activity or between different frequency bands throughout brain development^8,20,21^. Further, it is not well known how endogenous oscillations are modulated by exogenous stimuli during early development.

Previous studies of brain development have revealed a critical period when external experience is crucial for the normal development of cortical functional organization^22–24^. However, the development of functional organization may be independent of sensory experience, as suggested by recent studies showing that self-organized, spontaneous activity patterns precede and prepare for the emergence of maps^25–27^. Furthermore, a transient state of prolonged sensory responses was found in visual cortex but disappeared after eye-opening^4,21^. To untangle the influence of sensory-independent and experience-elicited neural activity on the development of cortical networks, it is necessary to compare patterns of network activity across different periods throughout development.

To investigate the link between patterns of neural activity and functional development, we recorded local field potential (LFP) and multiunit-activity (MUA) in the primary visual cortex (area 17) of juvenile ferrets throughout different stages of development. Ferrets, a species relatively immature at birth, are a well-established model system for studying neural development, with most previous studies focusing on functional organization^24,27–29^, or spiking activity^25,30–32^. Here, we focus on the time periods before and after eye-opening as a critical time point that separates periods dominated by endogenously generated and externally driven activity patterns, respectively. We focus on alpha/beta/low-gamma/high-gamma (8–15 Hz, 15–30 Hz and 30–55 Hz and 65–100 Hz, respectively) frequency bands due to their known role in coordinating spiking activity during cognition in the adult animal^33,34^. Our results reveal profound changes in high frequency oscillations after eye-opening, including (1) increase in high frequency (especially >30 Hz) oscillation amplitude, (2) decreased spike coupling to low frequency phase (theta/alpha) but increase to high frequency phase (>50 Hz), (3) decreased theta (4–8 Hz)/alpha/beta band amplitude coupling to delta (1–4 Hz) band phase but increased high-gamma band amplitude coupling to theta/alpha band phase, and (4) increased entrainment of the LFP by periodic exogenous visual stimuli. Our results support the hypothesis that the development of higher frequency oscillations (especially >30 Hz) serves as a biomarker for the maturation of cortical circuits.

## Results

**Figure 1:**
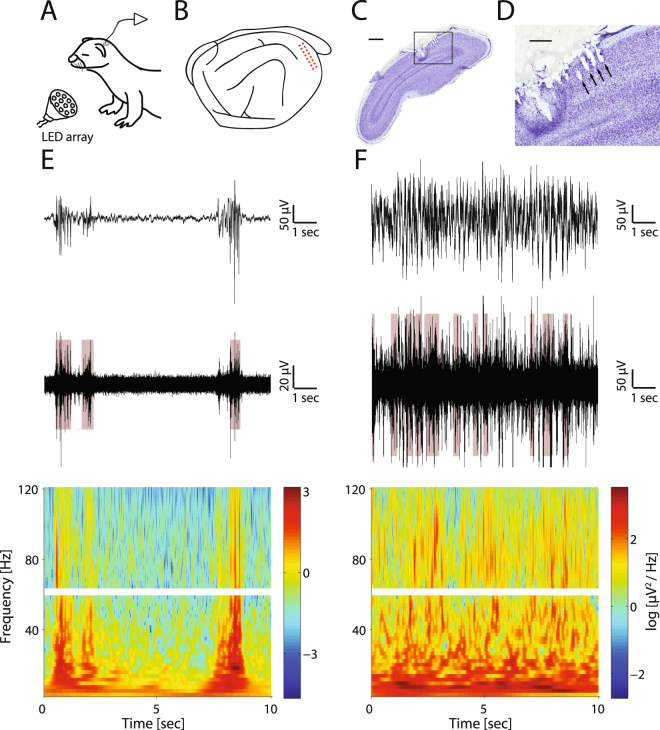
Experimental setup, recording locations and example spontaneous activity. (**A**) Experimental setup. Spontaneous or visually-elicited extracellular activity was recorded via implanted electrode arrays from awake, unrestrained juvenile ferrets. Visual stimulation was delivered using four LED arrays positioned in the four corners of the test cage. (**B**) Illustration of the 2 × 8 electrode array implant location in one example P25 animal brain. Each electrode is represented by a red dot. (**C**) Nissl stained section of V1 in a P40 animal showing the location of implanted electrodes. Scale bar represents 1 mm. (**D**) Close-up of the region enclosed by the black box in figure C. Black arrows indicate the electrode tracks. Scale bar represents 400 um. (**E**) Example spontaneous activity from an animal before eye-opening. From top to bottom: LFP trace (1–300 Hz), MUA (>300 Hz), and spectrogram of the same 10 second epoch. The brown highlighted epochs in the MUA plot represent the detected bursting periods. The spectrogram around 60 Hz is left blank due to the applied notch filter. (**F**) Example spontaneous activity from an animal after eye-opening. Same conventions and plot order as E.

**Figure 2:**
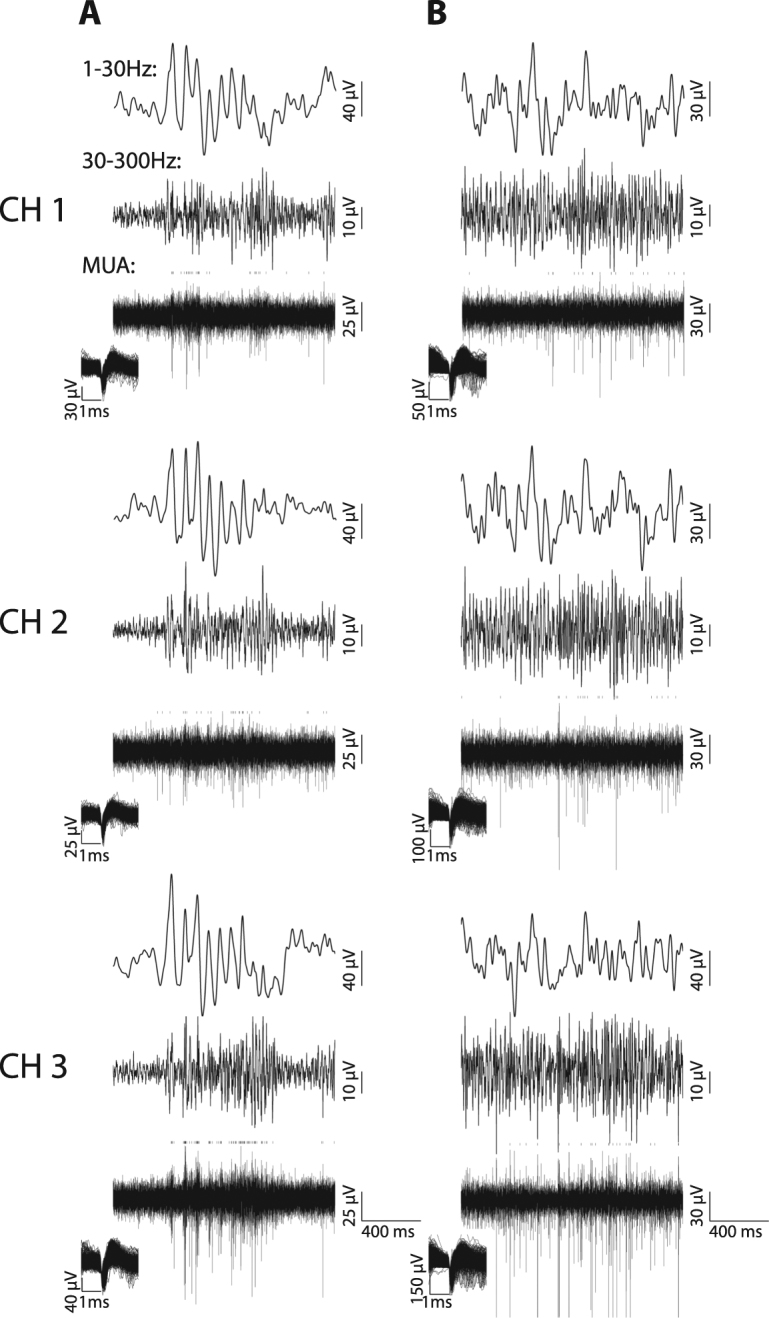
Additional example traces of spontaneous LFP and MUA before and after eye opening. (**A**) Example traces of three simultaneously recorded channels in another P29 animal. For clarity, raw signals were band-passed filtered and shown as low-frequency LFP (1–30 Hz, top in each subplot), high-frequency LFP (30–300 Hz, middle) and MUA (300–5000 Hz, bottom). Each short dashed line above the MUA traces indicates the detection of a spiking event. The insets in the bottom left of the MUA traces shows the shape of the detected spikes. (**B**) Example traces of three simultaneously recorded channels in another P43 animal. Same configuration as A.

We asked how both local- and network-level patterns of neural activity in ferret visual cortex change around eye-opening, a critical time point in development. To answer this question, we recorded spontaneous and visually-elicited MUA and LFP activity in visual cortex of freely-moving juvenile ferrets (Fig. 1A,B, see methods for details) before eye-opening (postnatal age P22-P29, mean ± SD: 26.0 ± 2.6, n = 9) or after (P33-P50, 41.3 ± 4.5, n = 11). All animals in the before eye-opening group had both eyes completely closed and all animals in the after eye-opening group had both eyes completely opened at the time of recording. We found that in contrast to the more continuous activity patterns presented after eye-opening (Fig. 1F, example from a P50 animal, also refer to Fig. 2 for more examples with higher temporal resolution), spontaneous activity before eye-opening was characterized by short periods of pronounced LFP amplitude and spiking rate (bursts), interleaved with periods of relative silence (Fig. 1E, example from a P29 animal). The alternation of bursts and periods of low-amplitude activity in ferrets before eye-opening is consistent with previous observations in the pre-term infant^13^ and rodents and ferrets before eye-opening^35–37^.

### Increase of high frequency oscillations after eye-opening

**Figure 3:**
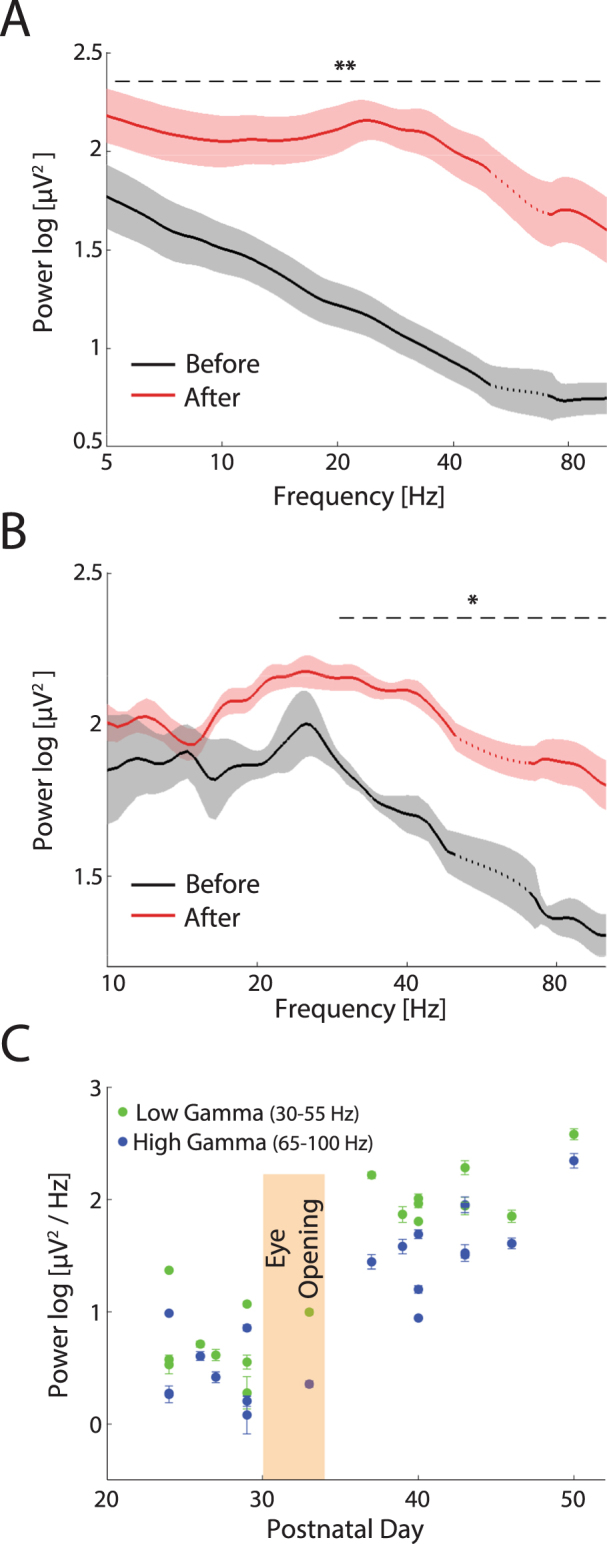
LFP power of spontaneous activity (animal in the dark) before and after eye-opening. (**A**) Power spectra (1/f normalized) across the whole recording session in animals before (black, n = 42 channels) and after (red, n = 102 channels) eye-opening. Traces and shadows represent mean and s.e.m, respectively. The data around 60 Hz (dotted lines) are removed and interpolated between adjacent data points due to the applied notch filter. The dashed line marks the frequency range in which the power is significantly different between the two periods. (**B**) Power spectra (1/f normalized) in burst periods in animals before eye-opening (black) and in firing-rate matched epochs after eye-opening (red). The dashed line marks the frequency range in which the power is significantly different between the two periods. (**C**) LFP gamma band power of spontaneous activity as a function of postnatal day. Green dots and blue dots indicate low gamma band (30–55 Hz) and high gamma band (65–100 Hz), respectively. Each dot represents data from one animal. Error-bars indicate s.e.m. The period of eye-opening is marked by the brown shaded box. Before eye-opening the gamma power was relatively stable and after eye-opening the gamma power increased. *p < 0.05, **p < 0.01.

To further quantify the difference in overall activity structure in these two periods, we compared the LFP power spectra and found that the power in all frequency bands was significantly increased after eye-opening (Fig. 3A, Mann-Whitney U-test, p < 0.001 for full frequency range, before eye-opening, n = 42 channels, after eye-opening, n = 102 channels). However, an increase in LFP power might merely be a consequence of the disappearance of the low-amplitude periods between bursts, which contribute little to the overall power. To control for this potential confound, we next compared LFP power during burst periods before eye-opening to LFP power after eye-opening. We defined the time windows of bursts as periods with a maximal inter-spike-interval of 100ms and minimal spike count of 10 (see Chiappalone *et al*., 2006^38^, also see Methods for details). This method efficiently identified the bursts before eye-opening (brown highlighted epochs in MUA trace in Fig. 1E). The same criterion was applied to data after eye-opening for comparison. Before eye-opening, the burst occurrence was 1.92 ± 2.40 bursts per minute, the average duration was 350.3 ± 155.8 ms, and the mean spike rate in burst periods was 64.32 ± 31.20 spikes/second. Applying the same criteria in activity after eye-opening yields higher occurrence (8.2 ± 9.5 bursts per minute, p < 0.001) and duration (447.5 ± 121.5 ms, p < 0.001), but lower intra-burst spike rate (42.64 ± 16.79 spikes/second, p < 0.001). Focusing on the LFP specifically within burst periods, we found that the power spectrum before eye-opening already exhibited multiple peaks (Fig. 3B, black line). However, in burst periods, power was lower before eye-opening compared to after eye-opening, and the differences were significant in the high-gamma band (50–100 Hz in Fig. 3B), indicating that the elevation of the overall LFP power after eye-opening resulted from both a decrease in duration of quiescent periods, and an increase of high frequency LFP amplitude in active periods. It is notable that besides the increase of absolute LFP power in all frequency bands, the relative power was enhanced in higher frequency bands (the percentage to total power: alpha, 8–15 Hz, Before = 4.59% ± 1.87%, After = 7.76% ± 3.13%, p < 0.05; beta, 15–30 Hz, Before = 2.95% ± 1.47%, After = 10.62% ± 7.55%, p < 0.05; low-gamma, 30–55 Hz, Before = 1.56% ± 0.96%, After = 6.43% ± 3.10%, p < 0.001; high-gamma, 65–100 Hz, Before = 0.95% ± 0.62%, After = 2.15% ± 1.12%, p < 0.05).

To investigate whether eye-opening is an important event in the development of high frequency oscillations, we plotted the low- and high-gamma power as a function of postnatal day across all animals. The results (Fig. 3C) revealed that gamma power dynamics were distinct for the two stages of development, and were separated by eye-opening: gamma power was at a stable low level before eye-opening; and increased gradually after eye opening. A Chow-test revealed that the linear regression coefficients in the two age groups were significantly different (low-gamma, before eye-opening slope = 0.002, n = 9 animals, after eye-opening slope = 0.062, p < 0.05, n = 11 animals; high-gamma, before eye-opening slope = −0.022, after eye-opening slope = 0.093, p = 0.05). Due to the increased level of visual stimulation upon eye-opening, these results indicate that external sensory stimuli may play an important role in the development of high frequency oscillations.

### Decrease of spike phase locking to LFP theta/alpha band after eye-opening

**Figure 4:**
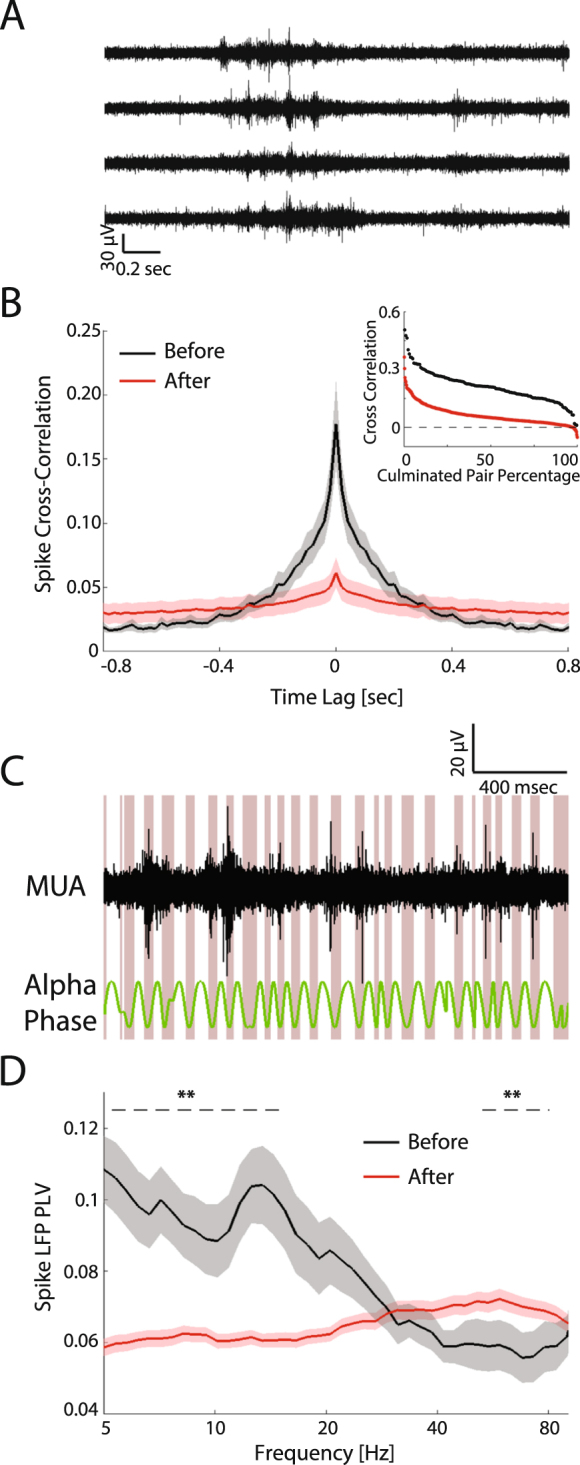
Synchronization and spike phase-locking of spontaneous activities before and after eye-opening. (**A**) Example MUA traces from four simultaneously recorded channels in an animal before eye-opening. (**B**) Main plot: spike cross-correlograms in animals before eye-opening (black, n = 7) and after eye-opening (red, n = 11). Traces and shadows represent mean and s.e.m, respectively. Inlet: distribution of the spike cross-correlation coefficients for each channel pair. Data are pooled from all animals and sorted by the correlation coefficient value. (**C**) An example MUA trace in which the spikes were phase-locked to the alpha/ low beta (8–20 Hz) cycle of the simultaneously recorded LFP. The troughs of the alpha/ low beta phase are highlighted by the red shading. (**D**) Frequency-resolved spike LFP phase-locking value before eye-opening (black, n = 27 channels) and after eye-opening (red, n = 96). Traces and shadows represent mean and s.e.m, respectively. The dashed lines mark the frequency range in which the phase-locking value is significantly different between the two developmental periods. **p < 0.01.

In order to determine if the observed differences in the temporal structure of the LFP impact neuronal spiking, we next studied the link between the LFP and the spiking activity (MUA). It is well-known that structured neuronal activity is crucial for circuit maturation in the visual system (e.g. retinal waves). In agreement with previous reports^37^ we observed that MUA was synchronized across channels before eye-opening (Fig. 4A for example of synchronized channels). We hypothesized that before eye-opening intrinsically generated network oscillations guide spiking activity, whereas after eye-opening spiking activity is less coupled to rhythmic patterns of network activity. Indeed, the representative trace in Fig. 4C shows that before eye-opening, spikes were highly phase-locked to the alpha LFP phase. This phenomenon of elevated spike-LFP phase locking before eye-opening corresponds to a peak in the alpha band in the phase-locking value curve (black line in Fig. 4D). However, after eye-opening the spike-phase locking was significantly decreased in the theta and alpha bands, but was increased in high gamma band (red line in Fig. 4D, p < 0.01 in 5–15 Hz and 50–80 Hz, before eye-opening, n = 27, after eye-opening, n = 96). The change of spike-phase locking was also accompanied with decreased MUA synchronization measured between electrode contacts (Fig. 4B, correlation coefficient value Before = 0.177 ± 0.088, n = 7, After = 0.061 ± 0.039, n = 11, p < 0.01, see Fig. 4B inlet for distribution of correlation coefficients for all channel pairs). This observation matches a previous finding that spontaneous activity exhibited decorrelation soon after eye-opening (Fig. 4B in red). It is also notable that negative correlation values were only found after eye-opening (Fig. 4B inlet). Therefore, in agreement with our hypothesis, the overall link between the LFP and MUA was altered after eye-opening. The decreased coupling to theta/alpha phase indicates the spiking activity is less influenced by low-frequency rhythms, while the increased coupling to the high-gamma phase hints at the development of the inhibitory component of the local network.

### Decrease of coupling of high frequency amplitude to delta phase

**Figure 5:**
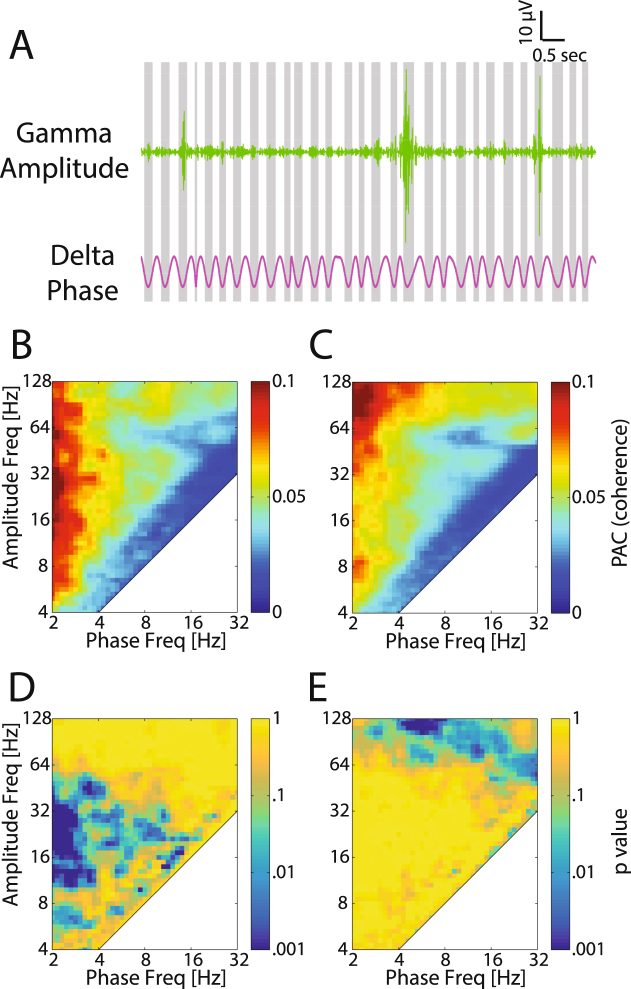
Spontaneous LFP phase-amplitude coupling before and after eye-opening. (**A**) An example trace of gamma (30–55 Hz) amplitude which was elevated at troughs of the LFP delta (2–4 Hz) cycle. Troughs of the delta oscillation are represented by gray shading. (**B**) Averaged phase-amplitude coupling before eye-opening (n = 9 animals). (**C**) Averaged phase-amplitude coupling after eye-opening (n = 11). (**D**) p value of Mann-Whitney U-test indicating whether phase-amplitude coupling is significantly stronger for before eye-opening compared to after eye-opening. The p values are without correction for multiple comparisons. (**E**) p value of Mann-Whitney U-test indicating whether phase-amplitude coupling is significantly weaker for before eye-opening compared to after eye-opening. The p values are without correction for multiple comparisons.

Given this changing link between oscillations and neuronal spiking, we next asked if the interaction of oscillations at different frequencies, which is known to be present in the adult animal, similarly changes with eye-opening. Considering the shift of the frequency in functional coupling between spike and LFP, we hypothesize that with eye-opening the coupling of alpha/beta/low-gamma amplitude to the phase of lower frequency oscillations also decreased. We tested this hypothesis by computing the phase-amplitude coupling of the LFP. An example trace recorded before eye-opening (P29, Fig. 5A) indicated that low-gamma band amplitude was modulated by delta phase. The averaged phase-amplitude coupling map shows that the amplitude of a broad band of LFP frequencies was modulated by the delta band (2–4 Hz) phase before eye-opening (Fig. 5B). After eye-opening, however, the amplitude coupling to the delta phase was decreased except for the high-gamma band (Fig. 5C), which was increased. High-gamma amplitude was also more coupled to theta band phase. Before eye-opening the cross-frequency phase amplitude coupling (PAC) value was significantly larger in theta/alpha/beta/low-gamma amplitude to delta-phase coupling, as well as in beta amplitude to theta/alpha phase coupling (Fig. 5D shows the p value indicating PACs were significantly decreased after eye-opening). In contrast, after eye-opening the PAC value was significantly larger in high-gamma amplitude to theta/alpha phase coupling (Fig. 5E shows the p value indicating PACs were significantly increased before eye-opening). Thus, these findings agree with the above result of a decreased overall functional coupling of rhythmic activity patterns upon eye-opening, and support a conceptual model in which the maturation of network dynamics in visual cortex is associated with an overall decrease in the integration of cortical rhythms at different frequencies (except in the high-gamma band).

We then investigated how fast the brain oscillations developed by looking at the spike-phase locking and phase-amplitude coupling in the first week after eye-opening, in which period the functional properties of the visual cortex develops quickly under the influence of visual experience^39,40^. We found that both the spike-phase locking and the phase-amplitude coupling rapidly transformed within the first week after eye-opening (Supplemental Fig. 1), suggesting a possible linkage between the development of neuronal synchronizations and the development of the functional properties^27^.

### Development of LFP amplitude, spike-phase locking and phase-amplitude coupling of visual-elicited responses

**Figure 6:**
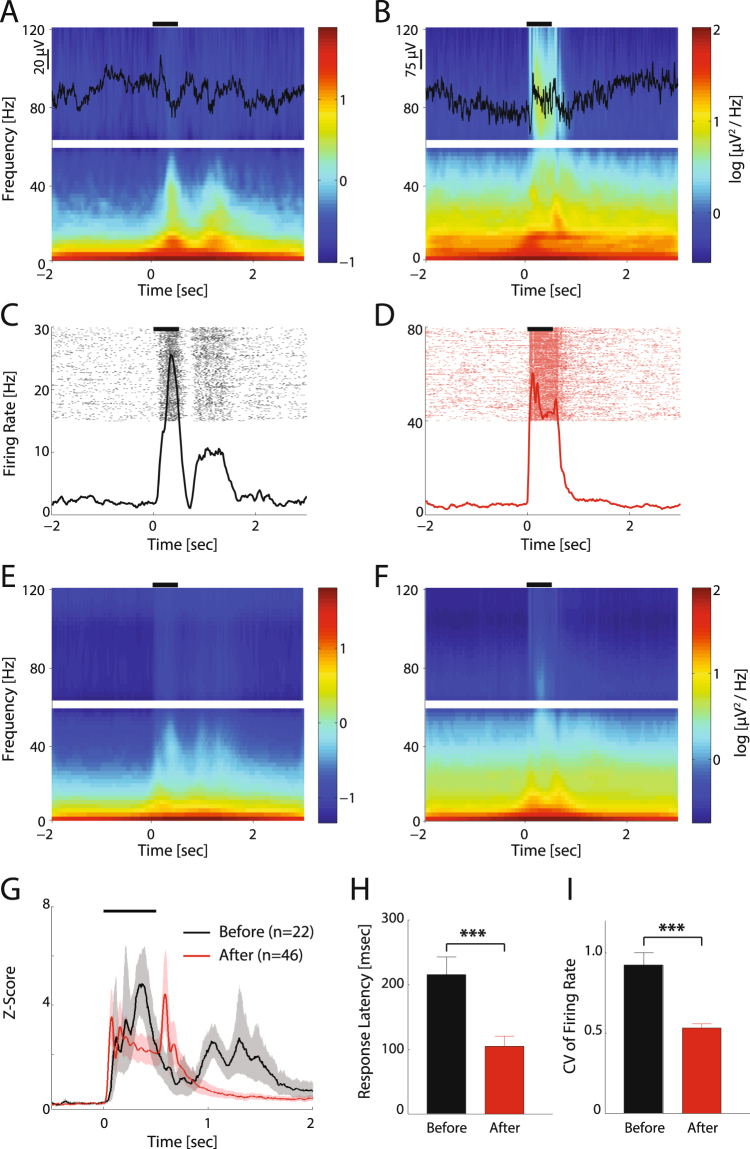
Visual responses before and after eye-opening. (**A,B**). Mean spectrograms of example recording session before (**A**) and after (**B**) eye-opening. LFP data (black traces) of one trial are superimposed on the top of spectrograms. The black lines above each spectrogram represent visual stimulus duration. The spectrogram around 60 Hz is left blank due to the applied notch filter. (**C**,**D**) Spike raster plots and peri-stimulus time histograms (PSTHs) from the same recording as A and B, respectively. Black lines above each plot represent visual stimulus duration. (**E**,**F**) Mean spectrograms across all recording sessions before (E, n = 7 animals) and after (F, n = 9) eye-opening. (**G**) Z-scored population PSTHs before and after eye-opening. Shaded regions represent 95% confidence intervals of the mean. (**H**,**I**) Response latency and coefficient of variance (CV) of firing rate (during the 500ms visual stimulation epochs) before (black) and after (red) eye-opening. Error-bar indicates s.e.m. ***p < 0.001.

We next asked if changes in spontaneous activity patterns are associated with similarly pronounced changes to sensory responses in cortex. While a detailed probing of visual responses is difficult in freely-moving juvenile animals, it has been previously established that visual stimuli evoke visual responses even before eye opening^4,21,30^. Specifically, we asked whether the visual cortex responds differently before and after eye-opening to bright 500 ms whole-field flash stimuli (See Methods). The flash stimuli elicited responses in visual cortex even before eye-opening (Fig. 6A for example LFP trace and spectrogram, 6 C for example spike raster and PSTH, 6E for population spectrogram and 6 G black trace for population PSTH), most likely via semi-transparent eye-lids^30^. Before eye-opening, response patterns were characterized by a second activity peak ranging from 500–1500 ms after stimulus offset, which were distinct from the response pattern confined to period of stimulus presentation after eye-opening (Fig. 6B,D,F, and red trace in Fig. 6G). Furthermore, the responses before eye-opening were also characterized by longer latency (Fig. 6H, Before: 215.2 ± 132.7 ms, n = 23; After: 104.8 ± 106.7 ms, n = 46, p < 0.001) and larger variance across trials (firing rate coefficient of variance, Fig. 6I, Before: 0.92 ± 0.38; After: 0.53 ± 0.18, p < 0.001). The prolonged responses after stimulus-offset, the higher response latency, as well as higher inter-trial firing rate variance indicates that the visual cortex was unable to respond to the sensory inputs in a temporally-constrained and stereotyped manner before eye-opening.

**Figure 7:**
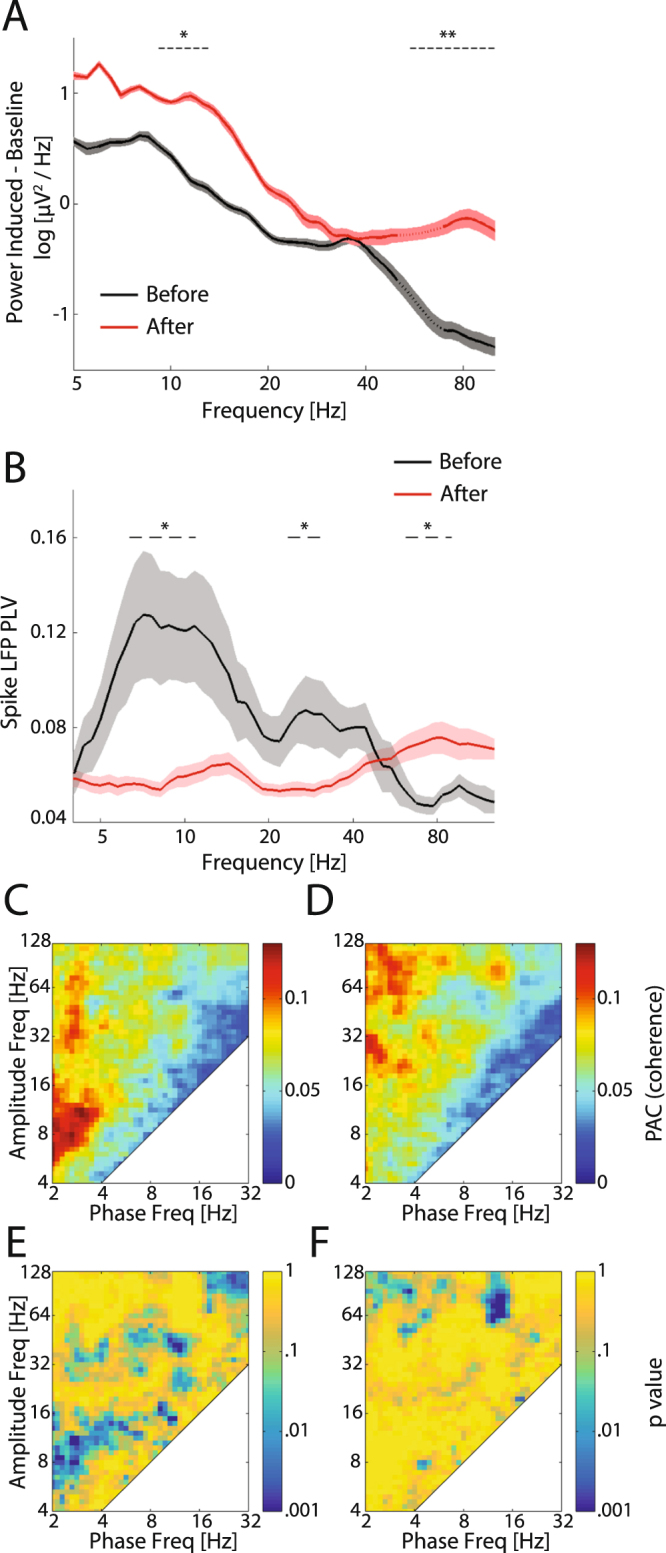
LFP power, spike LFP phase-locking, and phase-amplitude coupling of visually-elicited responses before and after eye-opening. (**A**) Power spectra of visually-elicited activity (subtracted from the baseline power) before (black, n = 7 animals) and after (red, n = 9) eye-opening. Traces and shaded regions represent mean and s.e.m, respectively. The data around 60 Hz (dotted lines) are removed and interpolated between adjacent data points due to the applied notch filter. The dashed lines mark the frequency ranges in which the power is significantly different between the two periods. (**B**) Frequency-resolved spike LFP phase-locking value before eye-opening (black, n = 13 channels) and after eye-opening (red, n = 43). Traces and shadows represent mean and s.e.m, respectively. The dashed lines mark frequency ranges in which the phase-locking value is significantly different between the two periods. (**C–F**) Phase-amplitude coupling of visually-induced oscillations before eye-opening (C, n = 7 animals) and after eye-opening (D, n = 9). P values indicate whether phase-amplitude coupling is significantly stronger (**E**) or weaker (**F**) before eye-opening compared to after eye-opening. *p < 0.05, **p < 0.01.

These changes in MUA responses prompted us to ask whether the visual-elicited LFP also changed after eye-opening. We hypothesized that the unit activity, which is less temporally-confined to visual stimuli is related to the immaturity of the inhibitory component of the neural circuit^35^, as reflected by lower amplitude high-frequency oscillations (especially gamma). Indeed, the spectrogram showed that visual stimuli elicited high-gamma (65–100 Hz) activation after eye-opening but not before (Fig. 6E versus 6 F). For a quantitative comparison, we calculated the difference of LFP power spectra between the baseline and visually-elicited responses (Fig. 7A). Before eye-opening, the LFP of visual responses had two local peaks at alpha and low-gamma (8–12 Hz and 30–40 Hz respectively as shown in the black line of Fig. 7A) but little activity above 40 Hz. After eye-opening, visual stimulation also elicited alpha activity, but the peak shifted to the high frequency band (~12–15 Hz as shown in the red line of Fig. 7A) compared to before eye-opening. In addition, higher-gamma activity was also substantially enhanced (40–100 Hz, p < 0.001, before eye-opening, n = 23, after eye-opening, n = 46). The overall visual responses were significantly higher after eye-opening in all frequency bands but, this increase was strongest in alpha/beta and high-gamma frequency bands. These results reveal that visual stimuli elicit larger amplitude high frequency LFP oscillations after eye-opening.

We then asked whether spike phase locking and phase amplitude coupling during visual responses develops in a similar way as observed for spontaneous activity. The results showed that, similar to patterns of the spontaneous activity, visual induced spiking activity was highly coupled to the low frequency LFP phase with two peaks in theta/alpha and beta bands, respectively. The phase locking value was significantly decreased in theta/alpha/beta bands but was increased in high-gamma band after eye-opening (Fig. 7B, p < 0.05, before eye-opening, n = 13, after eye-opening, n = 43). Accordingly, the phase amplitude coupling also shows similar changes from low frequency to high frequency: before eye-opening alpha amplitude was coupled to delta/theta phase, and low-gamma amplitude was coupled to theta/alpha phase (Fig. 7C,E shows PAC values which were significantly larger before eye-opening); while after eye-opening high-gamma amplitude was coupled to delta/alpha phase (Fig. 7D,F shows PAC values which were significantly larger after eye-opening). In summary, the spiking activity and LFP amplitude became less coupled to lower frequency phase and more coupled to higher frequency phase, suggesting the visual responses developed to be less synchronized by the slow intrinsic network rhythms but instead to be more synchronized by the higher-frequency network activity patterns that may be specific to the visual information processing.

### Enhanced entrainment of endogenous oscillations to high stimulus frequencies after eye-opening

**Figure 8:**
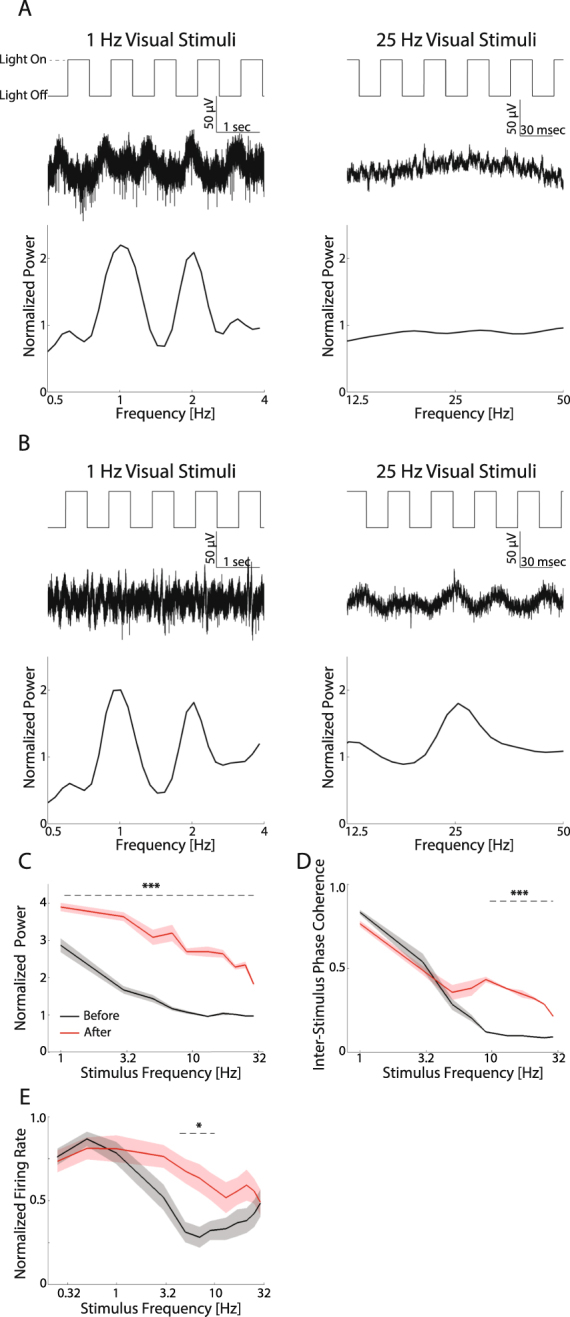
Visual entrainment to different stimulation frequencies before and after eye-opening. (**A**) Example neural responses to low frequency (1 Hz, left) and high frequency (25 Hz, right) visual stimuli from an animal before eye-opening. Top traces: visual stimuli pattern. Middle traces: broadband raw data from one channel. Bottom: Normalized power spectrum. Please note the time scales are different between the left and the right. (**B**) Example neural responses from an animal after eye-opening. Same configuration as A. (**C**) Normalized LFP power at the frequency of visual stimulation before eye-opening (black, n = 15) and after eye-opening (red, n = 9). LFP power was normalized by the session average. Traces and shadows represent mean and s.e.m, respectively. The dashed line marks the frequency range in which the LFP power is significantly different between the two periods. (**D**) Inter-stimulus phase coherence before eye-opening (black) and after eye-opening (red). Traces and shadows represent mean and s.e.m., respectively. The dashed line marks the frequency range in which the phase-locking value is significantly different between the two periods. (**E**) Normalized firing rate as a function of the stimulus frequency. Traces and shading represent mean and s.e.m, respectively. The dashed line marks the frequency range in which the firing rate is significantly different between the two periods. *p < 0.05, ***p < 0.001.

In the light of the increased high frequency oscillations in both spontaneous and visual-elicited LFP after eye opening, we further dissected the role of these oscillations in visual information processing. We hypothesized that visual responses before eye-opening were unable to reliably follow the temporal structure of visual stimuli, as the visual response was not time-constrained to the stimulus duration (Fig. 6A,C,E) and had less gamma power. To test this, we used a set of visual stimuli consisting of square-wave light flashes with frequencies ranging from 0.25–29 Hz presented in either ascending or descending order to evoke visual responses and investigate the capability of the network to be entrained by stimulus frequency. Example traces show that before eye-opening, oscillations were able to follow the pace of the visual stimulus only for low frequencies (Fig. 8A, left plots, note there was also a power peak at harmonic frequency), but not when the flashing was fast (Fig. 8A, right plots). This was not the case after eye-opening (Fig. 8B), when network activity reliably followed the temporal structure of all stimulation frequencies. In fact, the population-normalized LFP power at the stimulation frequency was near 1 before eye-opening when the stimulus frequency was above 10 Hz (black line in Fig. 8C), indicating the absence of LFP entrainment to visual stimuli above 10 Hz in this developing period. After eye-opening, the normalized LFP power at the stimulation frequency was increased (p < 0.001 for all frequency, before eye-opening, n = 15, after eye-opening, n = 9) for all frequencies tested. We further investigated whether this enhanced response power really reflected entrainment or just an overall increase of the evoked power, by looking at the inter-stimulus phase coherence of the LFP for flashes presented at each stimulus frequency. The inter-stimulus phase coherence was decreased as the stimulus frequency increased before eye-opening, with minimal inter-stimulus phase coherence observed for stimulus frequencies above 10 Hz (Fig. 8D). The inter-stimulus phase coherence for visual stimuli above 10 Hz was significantly increased after eye-opening (p < 0.001 for 9–29 Hz), indicating the elevated normalized power observed in Fig. 8C was at least partially the result of enhanced entrainment. The changes of visual responses to different stimulus frequencies were also reflected in spiking activity: the firing rate dropped when stimulus frequency increased beyond 3 Hz before eye-opening, but the frequency tuning was relatively broad after eye-opening (Fig. 8E, p < 0.05 for 5–9 Hz). Interestingly, we found a rebound of the firing rate in response to stimuli above 10 Hz before eye-opening. These results suggest that in development, the visual system increases its ability to capture the temporal structure of visual inputs, which coincides with the development of high frequency oscillations and desynchronization of the activity at lower frequencies.

## Discussion

The synchronization of populations of neurons reflected by the LFP has emerged as a fundamental mediator of brain function and behavior. In recent years, the development of the circuit-level mechanisms that enable neuronal synchrony have received growing attention. The present study recorded both MUA and LFP in the visual cortex in freely moving, juvenile ferrets. When comparing our recordings from after eye-opening to before eye-opening we found that (1) both spontaneous activity and visual responses were increased in the high-frequency band (gamma oscillations), (2) spike-locking to the theta/alpha band phase was decreased and to high-gamma band phase was increased, (3) coupling of theta/alpha/beta amplitude to delta phase was decreased while coupling of high-gamma amplitude to theta/alpha phase was increased, and finally (4) the LFP was more reliably entrained by higher frequency visual stimulation.

We found that the amplitude of high-frequency rhythms (gamma band) was increased after eye-opening. Previous results in humans^41^ and more recent reports in mice^12,20^ showed that the resting-state gamma oscillations continuously developed for a relatively long period during childhood, which has been suggested to correlate to both typical^42^ and atypical development^43^ of higher-order brain function. Our results reveal that gamma oscillations (both spontaneous and visually-elicited) increase from as early as immediately after eye-opening in freely-moving ferrets studied under more natural conditions without anesthesia or head-fixation. Furthermore, visually-elicited high gamma (65–100 Hz) oscillations also increased. The mechanisms underlying the change of high gamma band is awaiting future studies, as there are multiple synaptic or network processes as candidates (see review, Uhlhaas *et al*.,^44^).

We revealed that the amplitude of gamma band increased dramatically soon after eye-opening, indicating sensory experience may contribute to the development of gamma oscillations necessary for many cognitive functions. This notion is supported by a recent study that dark-rearing impaired the normal expression of beta/gamma oscillations^20^, probably by disrupting the development of inhibitory synaptic transmission^45^. However, sensory-independent factors, mainly the maturation of cortical networks, may play an essential role^46^. As a result, the continuously developing gamma oscillation observed in this study and in previous reports in human and mice^20,41^ may be distinct from the gamma oscillation observed in somatosensory cortex in neonatal rat^10^, which is driven by bottom-up thalamo-cortical input. Taken together, this result – when combined with previous reports - suggests that gamma and other high frequency rhythmic activity in the visual cortex starts to develop from an early stage (around eye-opening), probably by a combination of experience-related and sensory-independent mechanisms. Future studies are needed to investigate the time course of development of gamma oscillation in other cortical regions and its potential causal relation to the development of brain function.

One novel finding of the current study is the decrease of synchronization in early development (in the level of spike-field and inter-LFP-frequency coupling). We found strong spike-phase locking in theta/alpha band as well as theta/alpha/beta amplitude coupling to delta frequency rhythms before eye-opening, which may indicate high correlation of neuronal spiking^37^ (also see Fig. 4A). The synchronization decreased after eye-opening, as revealed by decreased spike-phase locking and phase-amplitude coupling in our results. This is in agreement with previous findings of decreased noise-correlation with circuit maturation in both electrophysiology^20,27^ and calcium-imaging studies^36,47^. Interestingly, the decrease of spike locking to theta/alpha phase and the decrease theta/alpha/beta amplitude coupling to delta frequency is accompanied by increase of spike locking to high-gamma phase and increase of high-gamma amplitude coupling to theta/alpha phase. Such increases were even clearer in visual-induced activities. The decrease of spike locking to theta/alpha phase and the decrease of theta/alpha/beta amplitude to delta phase may reflect the transition of the network activity from high synchronization, which facilitates synaptogenesis^48^ by Hebbian plasticity to a less synchronized mode^47^ for reducing neural noise to enable efficient information processing^49^, to boost local and global network communication^50^, as well as to develop dynamic and optimal internal models representing the environment^31,32^. The increase of gamma power, spike locking to the high-gamma phase, and high-gamma amplitude coupling to theta/alpha may benefit the grouping and selecting in visual information processing^51,52^, modulate synaptic plasticity^53,54^, or reflect the maturation of the cortical excitatory-inhibitory balance^55^.

Our study also revealed that the visual responses became more temporally refined and that the visual cortex increased its capability to follow higher stimulus frequencies after eye-opening. Brain oscillations in adult humans are synchronized by temporally modulated visual and auditory stimuli and exhibit a preferred resonance frequency in the gamma band^56,57^. In human developmental studies, these gamma steady-state responses increase through childhood and adolescence^58,59^. This enhancement was correlated to the desynchronization of the background activity^60^, indicating that the maturation of networks is necessary for supporting the steady-state responses observed in adults. Our results in the juvenile ferret align with the above human studies and indicate that the development of sensory entrainment of brain oscillations can start as early as immediately after eye-opening. Future studies will need to examine the presence of entrainment at higher frequencies than those tested in the current study, and to elucidate the development of the network mechanisms sustaining such steady-state responses^61^. It is also an open question if similar stimuli presented to probe the brain activity of preterm infants would reveal features of network activity that may predict subsequent developmental trajectories.

The functional properties of the ferret visual cortex undergo dramatic changes after eye-opening^26^. Weak orientation and direction selectivity already exist before eye-opening but visual experience is needed for their full development^39,40,62^. Compared to orientation selectivity, direction selectivity relies more on visual experience in the first few days after eye-opening for its development^39,40,62^. This difference could be explained by the need of developing inhibition mechanisms to robustly discriminate the motions in opposite directions^63–65^. Our data revealed improved temporal properties of visual responses after eye-opening (Figs 6 and 8). This result, combined with our finding that the changes of synchronization properties could happen within few days after eye-opening (Supplemental Fig. 1), illustrates a coincidence between the development of the neuronal synchronization and the development of the direction selectivity. Our findings also support a recent suggestion that simultaneously activating (by visual or optogenetic stimulations) of neuron population with similar initial direction preference will permit the further strengthen of the direction maps^66,67^.

Although the present research focuses on the changes of rhythmic activities in visual cortex after eye-opening, the observed results of oscillations and synchronizations after eye-opening may not be limited to the visual cortex, nor should the eye-opening be viewed as an isolated event in development that has implications only for the development of the visual system. Recent research shows that the opening of the eyelid is controlled by the BMP/SMAD signaling pathways^68^. Interestingly, the BMP/SMAD pathways also play an active role in cortical neuronal development^69^, suggesting that eye-opening is just one of the landmarks reflecting the continuous unfolding of the developmental plan. The plan, determined by the cascades of gene expression but also later shaped by intrinsic and externally-driven brain activity, may lead to the changes in oscillations and synchronizations in the visual cortex as well as in other cortical regions. The notion that the changes of cortical oscillations around eye-opening is a general maturational process across cerebral cortex is supported by the finding of increased gamma oscillation in the rat barrel cortex after eye-opening (Fig. 2D,E, plots after P15 in Minlebaev *et al*.^10^). However, the exact form of the oscillatory patterns and the time course of their development may be different across the brain as suggested by the inter-regional difference of the patterns and timing of development in ferrets^70^. The appearance of increased oscillations across brain regions after eye-opening in ferrets is coincident with the emergence of new motor skills and behaviors^71,72^.

Our results show the development of gamma oscillations around the time of eye-opening, indicating the possible involvement of sensory experience in this process. A recent study also found that dark-rearing after eye-opening reduced but did not completely abolish the development of gamma oscillations in rat visual cortex^20^. However, sensory-independent factors^46^ may also play a role. Specifically in ferrets, several mechanisms may participate in the change of oscillations and synchronization observed around eye-opening. First, the segregation into local circuits through clustering of local horizontal connections in cortex starts around one week before eye-opening; and clustering refinement finishes around one to two weeks after eye-opening in ferrets^73,74^. This refinement of connections may reduce the synchronization of the MUA and LFP. Second, the AMPA and kainate receptors in ferret visual cortex increase around the time of eye-opening, and NMDA receptors constantly increase for 2 months after birth^75^. The enhancement of glutamate receptor level may contribute the increased high frequency LFP power observed in the present study. Third, the balance of inhibitory/excitatory neurons in the ferret visual cortex^76,77^ as well as the inhibitory synaptic connections^78,79^ continues to develop after eye-opening up until approximately postnatal day 60. The resulting increase in synaptic inhibition may shape the presence of gamma oscillations, in which synaptic inhibition plays an important role, at least in the adult animal^80^. Fourth, although having arrived at the cortex before eye-opening, thalamic axons keep modifying their targeting in visual cortex in ferrets up to 7 weeks^81^, suggesting that the bottom-up thalamic influences on cortical oscillations and synchronization may continue to develop after eye-opening. Finally, the differences in behavioral state between the two groups examined here^82–84^, e.g., increased locomotion, may correlate with the strength of gamma oscillations. A comparison of spike-phase locking and phase-amplitude coupling between spontaneous activities and visually-induced responses (Figs 4D, 5B–D, 7B–F) suggests that the oscillations and synchronizations before and after eye-opening may be state-dependent; however assays such as pupillometry in head-fixed animals are needed to adequately address this question.

One limitation is that the current study did not explore activities in a broader developmental range. So it is not clear whether gamma oscillations have already developed in very early age in ferret visual cortex like in other species and other sensory cortex^10^. Further studies are also needed to address how gamma oscillations develop in visual cortex after 8 weeks old. Another limitation is that the current study did not specify how the oscillations across different cortical layers develop before and after eye-opening. Brain oscillations differ in different layers in both adult and neonates. Depth-probes will be needed to be deployed to look at the layer specific difference in development. Finally, it is still unclear what causes the increase of gamma oscillation and the change of frequency coupling after eye-opening. More casual studies are required, e.g., using visual deprivation to study the role of visual experience.

In summary, our results revealed a profound change of higher-frequency LFP activity during development in the ferret visual cortex. The study of LFP in ferret visual cortex around eye-opening may provide a useful tool for the study of cortical oscillations during both typical and atypical development.

## Methods

Seven pregnant ferrets (*Mustela putorius furo*) were acquired at the gestational age of 22–26 days from Marshall BioResources Inc. and housed individually until the delivery date (~E41). They were housed in a light cycle of 16 hr light/8 hr dark regimen throughout the pregnant and nursing periods to ensure the same breeding season cycle as maintained by the supplier. After birth, the kits were kept with their mothers (jills) from birth to the end of the experiment. Premium ferret diet (fat 15~20%, meat based protein 35~40%, Marshall Pet Products Inc., Wolcott, NY) was provided to the jills. The same food softened with warm water was used from 5 weeks of age to help wean the kits. A total of twenty kits (nine male and eleven female) were used in this study. All procedures were approved by the UNC – Chapel Hill Institutional Animal Care and Use Committee (UNC-CH IACUC) and in compliance with the guidelines set forth by the NIH (NIH Publications No. 8023, revised 1978) and USDA.

### Surgery

Electrode arrays were implanted in a brief surgical procedure one to three days before the electrophysiological recording session (recordings were performed at postnatal age P22-P50). Anesthesia was induced with four to five percent isoflurane in an induction chamber. Animals were maintained under anesthesia with 1.5%–3% isoflurane and 100% medical grade oxygen delivered through an anesthesia mask (David Kopf Instruments, Tujunga, CA). The depth of anesthesia was assessed by lack of toe-pinch response. Lidocaine (2%) was injected intramuscularly into the surgical site for local analgesia, and Furosemide (5%, 0.04 ml/kg) was injected intramuscularly to prevent cerebral edema. The electrocardiogram and breathing rate were monitored throughout the surgery to maintain deep general anesthesia (typical heart rate was 240 beats/minute and breathing rate was measured by a piezoelectric sensor). The body temperature was monitored with an infrared thermometer at least every 15 minutes. Hot-snap pads and a water heating blanket were used to maintain body temperature (36–38 °C) during surgery and subsequent recovery. Animals were fixed in a stereotaxic frame via ear bars (or ear cups if ear canals were closed) and a mouth bar (David Kopf Instruments). An initial scalp incision along the midline was made and a craniotomy was performed over primary visual cortex (area 17) located 1–3 mm anterior from lambda and 6–9 mm lateral from midline depending on animal age (Fig. 1B). The dura and pia were removed and a 2 × 8 electrode array (Innovative Neurophysiology, Durham, NC, 35 um tungsten electrodes with 200 um spacing, impedance of ~1 MΩs at 1 kHz, 0.5 mm shorter low impedance reference electrode) was lowered down into the cortex, oriented medial-laterally, until spikes or LFP signals were recorded. A silver ground wire was positioned on the surface of frontal cortex through an additional small craniotomy and was fastened to a nearby bone screw. The array was then fixed by dental cement which was anchored to the skull by bone screws. The incision site was sutured and triple antibody ointment was applied. Saline (10–20 ml/kg) warmed to body temperature was injected subcutaneously to prevent post-operative dehydration. After full recovery from anesthesia, the kit was returned to the litter. The body weight was measured twice a day for the following days to ensure proper recovery. Acetaminophen (Children’s Tylenol, 16 mg/kg) was administrated orally twice per day for at least 3 days after surgery for pain alleviation.

### Recording procedures and visual stimuli

Recordings took place in the housing facility in a 68.5 × 51.5 × 49 cm (L X W X H) ferret cage which was light-insulated by thick black curtains. A blanket or bedding was added to the floor to keep the animal warm and comfortable. The animal was allowed to freely move within the dark cage for 10–15 minutes while spontaneous neural activity was recorded via a light-weight, wired head-stage (Intan,Los Angeles, CA, RHD2132; Fig. 1A). A subset of the animals (16/20) were then presented with visual stimuli of light flashes presented from four computer-controlled LED arrays (220 Lumen each array) positioned in each corner of the cage. The duration of each stimulus was 500 ms with an inter-stimulus interval 4.5–7.5 seconds. Flash stimuli were repeated 100–200 times. Of all visually-tested animals, 6 were further tested with another pattern of visual stimuli consisting of flashes at different frequencies (chirp stimuli). These recording sessions started with 50 seconds of continuous 10 Hz flashes (square wave of 50% duty cycle). This block of 10 Hz served to habituate the visual system, and to avoid a strong onset response for subsequent stimulation. Then periodic flash stimulation at the following frequencies was presented in either ascending or descending order, 0.25, 0.5, 1, 3, 5, 7, 9, 13, 17, 21, 25, 29 Hz. Stimulation at each frequency was presented for at least 6 cycles or 3 seconds, whichever was longer. Both ascending and descending frequency stimuli were repeated 10 times, and were intermingled and randomized. Between two ascending/descending stimulation blocks, there was 20 seconds of 10 Hz conditioning stimulus. Some animals (6/20, not used in the chirp stimuli test) were also tested with an acoustic stimulus that consisted of 100 ms 70 dB DSL broadband noise delivered by two speakers on both sides of the cage. However, no auditory responses were detected in visual cortex (aged P25 - P43, data not shown). Each recording session was limited to approximately one hour. We did not use individual animals for longitudinal studies because of the fast expansion of brain volume and the resulting challenges of maintaining a chronic implant, especially before eye-opening^85^.

### Data collection

A light-weight wired headstage (Intan, RHD2132) connected to the implanted arrays amplified and digitized the neural signal (sampling rate of 20 kHz), which was transmitted via a cable to the main control board of the electrophysiology acquisition system (Intan, RHD2000). A camera (Microsoft LifeCam Cinema 720p HD Webcam) was modified to be infrared sensitive and was mounted on the cage ceiling to track the position of the animal. A data acquisition device (USB-6212, National Instruments, Austin, TX) was used to control the LED arrays for visual stimulation and an infrared LED mounted within the field of view of the camera. The data acquisition system simultaneously sent a periodic “on” pulse signal to the electrophysiology acquisition system and to the LED in order to synchronize video and electrophysiology data for post-hoc analysis.

### Histology

Immediately after recordings, animals were deeply anesthetized with an intramuscular injection of a ketamine/xylazine cocktail (40 mg/kg ketamine, 5 mg/kg xylazine). Electric currents (5 µA sine-wave, 10 second, by A395R Linear Stimulus Isolator, World Precision Instruments, Sarasota, FL) were passed between 2 to 3 sites on the multi-electrode array and the nearby reference electrode to produce lesions at the recording sites. The animal was then humanely euthanized with an overdose of sodium pentobarbital and immediately perfused with 0.1 M PBS followed by 4% formaldehyde. The brain was extracted and post-fixed for at least 4 days in 4% formaldehyde before being transferred to 30% sucrose. After sinking in sucrose, the brains were then cut into 50 µm sections using a cryostat (VT-1200, Leica Microsystems, Wetzlar, Germany) and processed for Nissl stainingto confirm the electrode tracks (Fig. 1C,D). These lesions were larger than the scale of the electrode size, which occurred when removing the array from the brain, and were exaggerated by subsequent histological processing.

### Data analysis

Data were analyzed using custom-written scripts in Matlab (Mathworks, Natick, MA). Only data sections validated by video to be free of motion artifacts and in which the animal was awake (e.g. when body movements were observed) were used for further analysis. The raw data were notch-filtered at 60 Hz to remove line noise. A low-pass or high-pass filter with 300 Hz cut-off frequency (2^nd^ order butterworth filter with the zero-lag) was used to extract LFP and MUA signals, respectively. The LFP spectrogram was computed by convolving LFP signals with a family of Morlet wavelets (0.5 to 120 Hz in 0.5 Hz steps). The mean power spectrum either across the whole recording session or in the burst periods (see below for the definition of burst periods) was estimated by averaging the square of the absolute value of the convolved signal across the time of interest. However, such estimation for low frequencies may be inaccurate if the time window is short. Thus, we only present results of the analysis on frequencies larger than 10 Hz for the power spectrum in burst periods. To account for the power law scaling of the LFP power spectrum, the power spectra were 1/f normalized by multiplying the each data point with its frequency. Spikes were extracted using a threshold of minus-five-times the standard deviation of the high-pass filtered signal. We used the method of a previous study^38^ to detect the burst events in neural data before eye-opening. Briefly, the inter-spike-interval (ITI) was calculated from the spike train of each spontaneous recording session (before eye-opening, ISI = 499.6 ± 1618.7 ms, mean ± SD; after eye-opening, ISI = 132.3 ± 418.8 ms). A burst was defined as a period within which: (1) the maximal ISI was less than 100 ms, and (2) a minimum of 10 spikes were counted.

To calculate spike cross-correlation, we computed the cross-correlogram of two spike trains binned into 20 ms windows. The lag time ranged from −250 ms to 250 ms with step length equals to the bin width. We found that for almost all channel-pairs, the peak of the cross-correlogram was at zero. Therefore, spike correlations were defined as the correlation coefficient of spike times. We also calculated the results using smaller bin widths of 5 ms or 2 ms and found that the choice of the bin width did not affect our finding.

To compute the phase-amplitude coupling and spike-field coherence, we convolved the LFP signal with a family of complex Morlet wavelets (2 to 128 Hz, with logarithmic step length of 2^1/8^). The resulting signal was then used to estimate the instantaneous phase and amplitude LFP signals within specific frequency bands. To estimate the strength of spike locking to the phase of LFP oscillations, we computed the spike phase locking value (PLV)^86^. Spike PLV was defined as

1 $$PL{V}_{spk}({f}_{0})=\frac{1}{N}|\sum _{n=1}^{N}{e}^{i{\theta }_{n}^{spk}({f}_{0})}|$$

where ${\theta }^{spk}$ is the instantaneous LFP phase at frequency *f*_0_ at the time of spiking activity. This analysis projects the LFP phase at the time of each spike onto a unit circle. The magnitude of the mean of all unit vectors gives an indication of the strength of spike phase locking, with values close to 0 indicating no phase locking, and values close to 1 strong phase locking.

To control for the effect of different firing rates in different age groups on computing the spike-phase locking, we used a bootstrap method to estimate the spike PLV controlled for firing rate: 400 spikes were randomly selected with repetition to compute spike PLV. The same procedure was then repeated 5000 times and the mean resulting firing rate unbiased spike PLV was computed. To ensure the reliable estimation of spike PLV, channels where less than 400 spikes were detected for each recording session were discarded. To control for spike waveform contamination of the LFP signal, especially on high frequency components, we used the neighboring channel to estimate LFP phase for spike PLV analysis. Cross-frequency phase amplitude coupling (PAC) was computed to assess the degree with which high frequency oscillations are temporally organized by the phase of low frequency oscillations. PAC was defined as the phase locking value between a low frequency signal and amplitude fluctuations of a high frequency signal occurring at the lower carrier frequency. First, an LFP signal *x*(*t*) was convolved with a Morlet wavelet with carrier frequency *f*_1_

2 $$X(t,{f}_{1})=x(t)\,\ast \,w(t,{f}_{1})$$

where * denotes the convolution operation and *w*(*t*, *f*_1_) a Morlet wavelet with center frequency *f*_1_. Then, the analytic amplitude of the same signal *x*(*t*) at a higher carrier frequency *f*_2_ was convolved with a Morlet wavelet at the lower carrier frequency *f*_1_.

3 $$X(t,{f}_{2})=|x(t)\,\ast \,w(t,{f}_{2})|\,\ast \,w(t,{f}_{1})$$

where *X*(*t*, *f*_2_) captures fluctuations in the amplitude of the high frequency signal that occur at the lower carrier frequency. The PAC between carrier frequencies *f*_1_ and *f*_2_ is then defined as the phase locking value between *X*(*t*, *f*_1_) and *X*(*t*, *f*_2_).

4 $$PAC({f}_{1},{f}_{2})=\frac{1}{N}|\sum _{n=1}^{N}{e}^{i({\theta }_{n}^{diff})}|$$

where ${\theta }^{diff}$ represents the phase lag between *X*(*t*, *f*_1_) and *X*(*t*, *f*_2_).

For visual responses, spectrograms were computed per trial using wavelet convolution as described above, and then averaged across trials. The power spectrum of the elicited LFP was computed by averaging the spectrograms in the visual stimulus periods. The baseline power spectrum was estimated by averaging the spectrograms rate in the baseline period (500–1000 ms before stimulus onset). To compute the spike-phase locking and phase-amplitude coupling of visual responses, we used induced LFP (raw LFP subtracted by the event evoked potential on a trial-by-trial basis) to compute the phase and amplitude. We also used trial-shuffling to control the effect of phase reset by the visual stimulus. However, we found that subtraction of evoked potentials was sufficient to remove nearly all of the phase reset effect.

Peri-stimulus time histogram (PSTH) was made using a 100 ms smoothing window. The PSTH was then z-scored by subtracting the mean firing rate in the baseline period then dividing by the standard deviation. The population PSTH was then computed by averaging across all units. The 95% confidence interval was estimated using a bootstrap procedure on the individual PSTHs with 10,000 repetitions.

Visual response latency was estimated using a previous method^87^. Briefly, after binning the spike counts in 1ms bins, we fitted the Poisson distribution parameter of the baseline period (500 ms immediate before stimulus onset) across all bins and all trials. The response latency was taken as the first bin after stimulus onset that: (1) exceeded a P = 0.01 difference from background distribution and (2) the subsequent 2nd and 3rd bin exceeded a P = 0.05 level. The method relies on the accurate estimation of baseline distribution, thus it works well with a large number of trials (100 and more) as in the present study.

Visual response variance across trials was characterized by coefficient of variance (CV), defined as the standard deviation of the firing rate in 0–500 ms after stimulus onset divided by the mean firing rate during the same time period.

To study the response to the ‘chirp’ stimuli, the spectrogram was normalized by the power spectrum computed across the whole recording session. We tested the entrainment of endogenous oscillations to externally applied visual stimulation using two complementaryapproaches: First, we computed the normalized LFP power at the stimulation frequency. Note that this first method is independent of the phase relationship between endogenous oscillations and the applied stimulus. Second, we computed the inter-stimulus phase coherence for flashes presented at each stimulus frequency. This analysis quantifies how well endogenous LFP oscillation phase is entrained by the applied rhythmic stimulus.

Unless otherwise specified, all results are calculated from the signals of the recording channels that exhibited clear spiking activity. Results and statistics of LFP results (power spectrum and cross-frequency phase amplitude coupling) are computed based on animal number. Other MUA based results are calculated by channel numbers. The average number of channels per animal is 4.67 ± 4.27 (Mean ± SD, range 1–15, n = 9 animals) before eye-opening, and 9.27 ± 5.22 (range 3–16, n = 11) after eye-opening for recordings of spontaneous activity. For analyzing the results of visual responses we further excluded the channels in which the spiking activity was not modulated by the visual stimuli. To this end, we tested whether the firing rate during visual stimulation was significantly different from the baseline firing rate recorded 0–500 ms before stimulus onset using two-tailed student t-test with α = 0.05. As a result 67.7% (23/34) of the channels before eye-opening and 66.7% (46/69) of the channels after eye-opening were included. The average number of channels for analyzing visual responses were 3.29 ± 3.55 (Mean ± SD, range 1–11, n = 7 animals) before eye-opening, and 5.11 ± 4.94 (range 1–13, n = 9) after eye-opening. All statistics are presented as mean ± standard deviation unless otherwise specified. All the significance tests were performed using the Mann-Whitney U-test. The Holm-Bonferroni method was used to control for multiple comparisons. To control for the multiple-comparisons for the frequency resolved results (power spectrum and spike LFP phase locking), we first determined neighboring frequencies that did not overlap (Morlet wavelets are Gaussian shaped in the frequency domain leading to dependence of similar frequencies). Specifically, we define adjacent non-overlapping frequencies as those whose central frequencies are separated by at least two standard deviations in the frequency domain. As a result, frequencies equally spaced in half an octave were selected. We then selected p-values for these frequencies to reduce the dependence among adjacent frequency points. Then the Holm-Bonferroni method was used to control for multiple comparisons, and frequency ranges that passed the significance level were indicated.

The datasets generated during and/or analyzed during the current study are available from the corresponding author on reasonable request.

## Electronic supplementary material

Supplemental Figure 1.
