# Supplementary material for: Early Development of Network Oscillations in the Ferret Visual Cortex
Source: Sci Rep. 2017 Dec 19;7:17766. doi: 10.1038/s41598-017-17502-y (PMC5736753; doi:10.1038/s41598-017-17502-y)
Supplement: Supplementary file 1 — Supplemental Figure 1. [file 41598_2017_17502_MOESM1_ESM.pdf]

Supplementary information for:

Early Development of Network Oscillations in the Ferret Visual Cortex

Yuhui Li[1], Chunxiu Yu[1], Zhe Charles Zhou[1,2], Iain Stitt[1], Kristin K. Sellers[1,2], John H. Gilmore[1], and Flavio Frohlich[1,2,3,4,5,6]

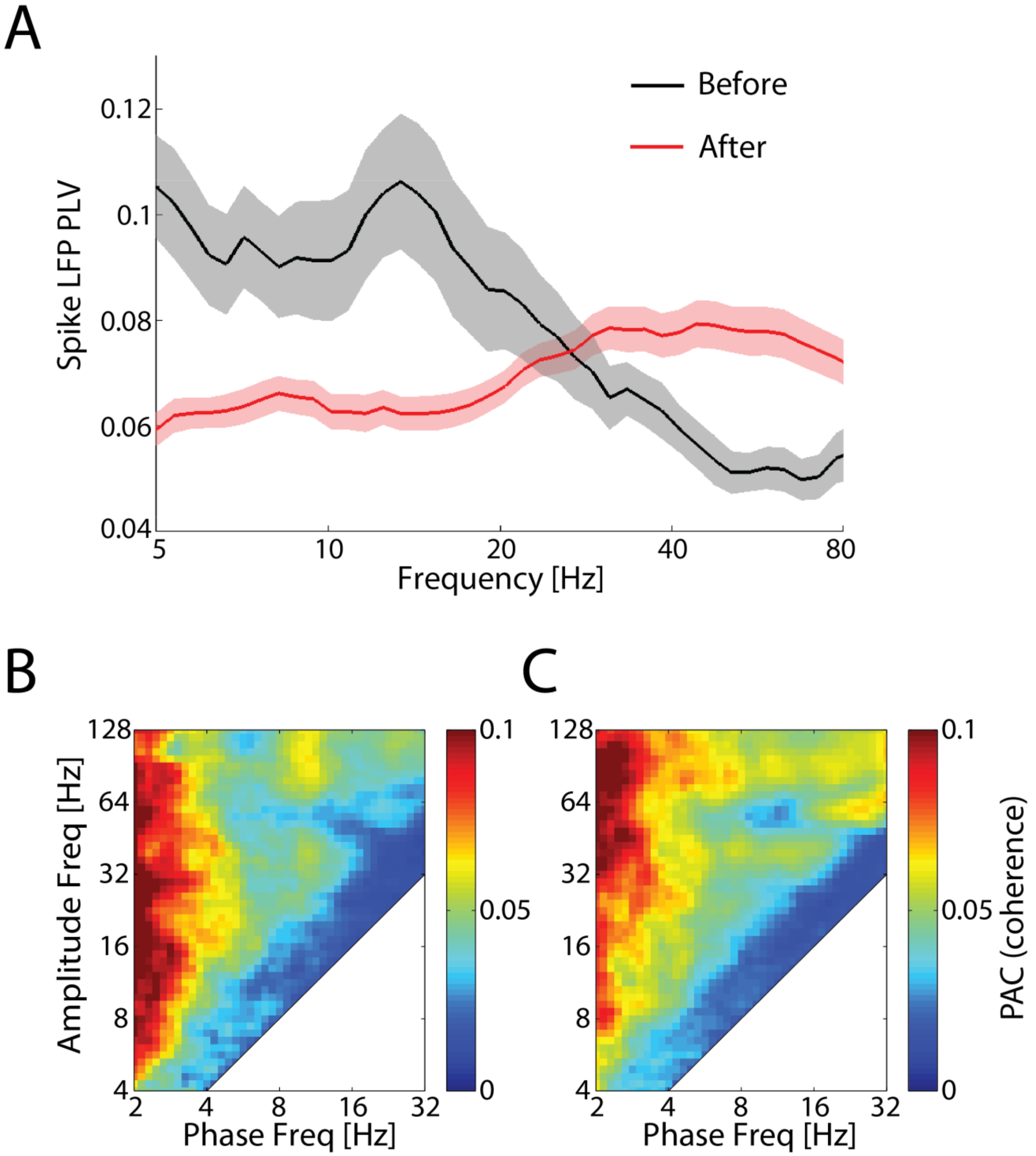

A
Before
After
Spike LFP PLV
0.12
0.1
0.08
0.06
0.04
5
10
20
40
80
Frequency [Hz]
B
C
Amplitude Freq [Hz]
128
64
32
16
8
4
2
4
8
16
32
Phase Freq [Hz]
0.1
0.05
0
PAC (coherence)

Supplemental Figure 1. Spike phase-locking and phase-amplitude coupling of spontaneous activities one week before (P24-P29) and one week after (P33-P38) eye-opening.

A. Frequency-resolved spike LFP phase-locking value one week before (black, n = 23 channels) and one week after eye-opening (red, n = 7). Traces and shadows represent mean and s.e.m, respectively.

B. Averaged phase-amplitude coupling one week before eye-opening (n = 6 animals).

C. Averaged phase-amplitude coupling one week after eye-opening (n = 2 animals).
